# Validity and reliability of the Mandarin version of Patient Dignity Inventory (PDI-MV) in cancer patients

**DOI:** 10.1371/journal.pone.0203111

**Published:** 2018-09-06

**Authors:** Yu-Chi Li, Hsiu-Hung Wang, Chung-Han Ho

**Affiliations:** 1 Department of Nursing, Chung Hwa University of Medical Technology, Tainan, Taiwan; 2 College of Nursing, Kaohsiung Medical University, Kaohsiung, Taiwan; 3 Department of Medical Research, Chi Mei Medical Center, Tainan, Taiwan; 4 Department of Pharmacy, Chia Nan University of Pharmacy and Science, Tainan, Taiwan; University of Queensland, AUSTRALIA

## Abstract

Nurses play an important role in maintaining patients’ dignity. How to measure patients’ dignity and dignity-related distress is an important issue in nursing care. Use of a reliable and valid tool assessing dignity-related distress in patients is necessary. The study investigated the validity and reliability of the Mandarin Version of the Patient Dignity Inventory (PDI-MV) in cancer patients. The Patient Dignity Inventory (PDI) was translated into the Mandarin language using forward and back translation. A convenience sample of 125 adult cancer patients was recruited from the oncology ward of a medical center in southern Taiwan. Factor analysis with principal axis factoring extraction method and oblique rotation (promax) was used to assess the construct validity. Concurrent validity was established using the Patient Health Questionnaire-9 (PHQ-9), Mandarin version of Demoralization Scale (DS-MV) and the Rosenberg Self-Esteem Scale (RSES). Internal consistency was used to examine the reliability. Data were collected from February to May 2016. As a result of the factor analysis, four factors, including existential distress, loss of support and sense of meaning, symptom distress, and loss of autonomy. Concurrent validity showed that the PDI-MV was significantly correlated with the PHQ-9 (*r* = 0.25–0.54), DS-MV (*r* = 0.30–0.58) and the RSES (*r* = - 0.08 to—0.30), Cronbach’s alpha coefficients for the PDI-MV and four factors were 0.95, 0.95, 0.84, 0.83, and 0.89 respectively. The PDI-MV is a psychometrically sound instrument assessing a broad range of dignity-related distress issues in cancer patients.

## Introduction

Dignity is an inherent, intrinsic characteristic of humans that can be demonstrated by actions showing a respect for oneself and others [1]. Dignity can be classified as basic or absolute dignity and personal or relative dignity [1, 2]. Basic or absolute dignity is a universal concept referring to an intrinsic moral value present in everyone; it is related to an individual’s value, freedom, responsibility, and ability. By contrast, personal or relative dignity is constructed by society, and is the result of interactions between human beings; in other words, it is a dignity granted by the roles constructed by society [1–3]. Dignity is also identified as human dignity, which is a universal value of the human race, and social dignity, that is the product of the interaction between individuals and society, culture and tradition [4].

Many studies have pointed out that medical institutions were risk factors to damage patients’ dignity due to the vulnerable conditions of patients [5–7]. Previous literature has also pointed out that experience of patient-perceived dignity could explain the mutual relationship between health status and human rights [6, 7]. Preserving patients’ dignity represents the key concept for good healthcare provider practices in medical institutions [5, 6, 8]. Several studies have shown that preserving patients’ dignity could promote the sense of well-being, increase compliance of treatments, and reduce the rate of hospitalization repeats and dependence [9, 10]. Preserving patients’ dignity is not only a moral obligation for health care providers and a major ethical consideration, but is also intrinsic to nursing care [3, 11, 12]. Recently, the importance of patient’s dignity preserving care has been approved worldwide [13]. Several studies have focused on patients’ dignity-related distress experiences, such as the low sense of dignity, desire for death, demoralization, depression, and anxiety [14, 15]. Patients’ dignity-related distress issues are also importance in nursing and have been referred to nursing courses worldwide [16]. Several studies have posited that nurses play the most important role in maintaining patients’ dignity [16, 17]. Dignity preservation is essential for patients, but it remains a subjective concept without clear definition or explanation; furthermore, there is a general lack of clinical tools for assessing the dignity of patients in clinical settings. Thus, how to measure patients’ dignity is considered a difficult issue by nurses or healthcare providers. Although the previous studies have shown the prevalence of dignity-related concerns among the various associations between dignity and common sources of distress, we need a better understanding of how patients perceive dignity and dignity-related issue.

In 2002, Chochinov *et al* constructed an empirically based model dignity in the terminally ill [18–20], which was used in health systems. The Dignity Model consisted of 3 main categories: (1) ill related concerns, (2) dignity conserving repertoire, (3) social dignity inventory, include a broad range of issues, such as physical, psychosocial, spiritual, and existential. These issues may influence a dying patient's sense of dignity and dignity-related distress. To measure these issues, Chochinov *et al* developed the Patient Dignity Inventory (PDI) in the terminally ill [15]. The PDI has been translated into various languages, such as Persian, Italian, Spanish, and German [21–24], and the psychometric properties of these versions have been assessed. Cronbach’s alpha coefficients for Persian version was 0.85 [21], Italian version was 0.96 [22], Spanish version was 0.89 [23], and the German version was 0.96 [24]. By the PDI, we can understand patients' dignity- related distress. The original English version of the PDI comprises 25 items with five factors: symptom distress, existential distress, dependency, peace of mind, and social support. The factor of symptom distress comprises the PDI items that essentially cover physical as well as psychological sources of distress; the factor of existential distress is significantly correlated with various measures of psychological distress, quality of life, and suffering; that of dependency includes not being able to perform the task of daily living, not being able to attend to bodily functions and reduced privacy; that of peace of mind includes concerns about one’s spiritual life; and that of social support includes not feeling supported by friends, families, healthcare providers, and not being treated with respect [15]. Each item of the PDI is rated on a scale of 1 to 5 (1 = “not a problem,” 2 = “a slight problem,” 3 = “a problem,” 4 = “a major problem,” and 5 = “an overwhelming problem”). The total score ranges from 25 to 125, and a higher score indicates a lower level of dignity. Regarding its reliability and validity, the Cronbach’s alpha coefficients were 0.93 for the overall PDI, and 0.80 for symptom distress, 0.83 for existential distress, 0.77 for dependency, 0.63 for peace of mind, and 0.70 for social support. The test-retest reliability coefficients were 0.85 for the overall PDI and 0.37 to 0.76 for each item. Furthermore, the five subscales showed significant negative correlations with the Beck Depression Inventory (*r* = 0.17–0.38, p < 0.001) and Quality of Life Scale (*r* = -0.10 to -0.28, p < 0.001) [15]. Thus, overall, it has satisfactory validity and reliability.

Previous study reported that 87.1% of cancer patients felt ‘not being treated with respect’ would have a profound influence on their sense of dignity [25]. Cancer patient’s sense of dignity had significant correlation with depression, demoralization, well-being and quality of life [26, 27]. Understanding the associated risk factor of distress and sense of dignity is vital to field of cancer patients. The PDI was able to expose various aspect cancer patients’ distresses, covering a broad range of concerns [26]. In view of the previous literature and the PDI was originally tested on cancer patients’ dignity, the purpose of the study was to investigate the reliability and validity of the Mandarin Version of the Patient Dignity Inventory (PDI-MV) in cancer patients. In further research, this questionnaire could be used in Mandarin-speaking countries.

## Materials and methods

The study protocol received approval from the Institutional Review Board (IRB) of Chi Mei Medical Center (IRB number: 10411–003)

### Participants

A cross-sectional, validity and reliability assessment study was conducted. A sample of cancer patients was recruited from an oncology ward of a medical centre in Southern Taiwan. The inclusion criteria were: (1) aged over 20 years; (2) inpatients at any stage of cancer; (3) mentally alert, clear, and able to communicate in Mandarin; and (4) able to express their own opinions and complete the questionnaires. The exclusion criteria were (1) unconscious and (2) having organic diseases of the brain as diagnosed by a physician. For factor analysis, according to “the rule of 5” concerning the subjects-to-variables (STV) ratio, the minimum sample size was estimated by the number of subjects larger by five times the number of variables [28]. The original English version of the PDI contains 25 items, so the sufficient sample size in this study was estimated as 125. Data were collected from February to May 2016.

### Measures

#### Demographic and clinical background information

The structured questionnaires used to collect data comprise demographic characteristics (i.e., gender, age, marital status, education, monthly income, cohabitation status, and religious beliefs) and disease characteristics (i.e., tumor site, cancer stage, clinical characteristics, and treatment).

#### The Patient Health Questionnaire-9 (PHQ-9)

The PHQ-9 comprises two parts: one assessing depressive symptoms and one assessing functional impairment in daily life. The PHQ-9 assesses the degree of distress over the past two weeks, with each item being rated on a scale of 0 to 3. The total score ranges from 0 to 27. The total scores can be classified as follows: 0 to 4 = minimal depression, 5 to 9 = mild, 10 to 14 = moderate, 15 to 19 = moderately severe, and 20 to 27 = severe. The Cronbach’s alpha for the English version was 0.86 to 0.89 and the test-retest reliability was 0.84 [29]. In a previous study, the Cronbach’s alpha for the Mandarin version was 0.80, and the test-retest reliability was 0.87 [30].

#### The Mandarin version of Demoralization Scale (DS-MV)

The DS-MV [31] comprises 24 items in five factors (loss of meaning, dysphoria, disheartenment, helplessness, and sense of failure). Each item is rated on a scale from 0 to 4. The total score ranges from 0 to 96, with a score of over 30 indicating high demoralization. The Cronbach’s alphas of the overall scale and five factors (loss of meaning, dysphoria, disheartenment, helplessness and sense of failure) were 0.92, 0.84, 0.69, 0.88, 0.72, and 0.63, respectively. In terms of the validity, a negative Pearson’s correlation was found between the DS-MV and the Mandarin version of the McGill Quality of Life questionnaire (*r* = - 0.68, p < 0.001), while a positive correlation was found in the Mandarin version of the Beck Hopelessness Scale (*r* = 0.70, p < 0.001) [31]. This indicates that the DS-MV provides criterion validity.

#### The Rosenberg Self-Esteem Scale (RSES)

The RSES, a measure of self-esteem, was developed by Rosenberg in 1965. This inventory has been employed worldwide. It comprises 10 items, each scored on a scale from 1 to 4; the total score ranges from 10 to 40, with higher scores indicating higher level of self-esteem [32]. A number of studies in Taiwan have employed the Mandarin version of the RSES, and have reported Cronbach’s alphas above 0.80 [33–36]; thus, the scale has satisfactory reliability.

### Translation procedure and data collection

The PDI was dveloped by Chochinov [15]. The PDI was first translated from English into Mandarin separately by two nursing experts with PhD degrees who had studied in the United States. The translations were discussed with and modified by the authors until everyone agreed on a final version. Then, a nursing scholar proficient in both English and Mandarin back-translated the Mandarin version into English and confirmed the Mandarin translation completely fit with the original meanings of the English version. The Mandarin versions of the items and the back-translation were authorized by Chochinov.

After translation process, we began work on confirming the validity and reliability of the PDI-MV. Regarding the content validity, two nursing experts with rich experience in cancer care, one head oncology nurse, one palliative care physician, and one oncologist assisted in the evaluation of the content of the PDI-MV. The content validity index (CVI) was used to measure the content validity [37]; it was calculated by having experts rate each item on the scale, as follows: 1 = “very unsuitable,” 2 = “unsuitable,” 3 = “suitable after modification” and 4 = “suitable.” After summing the scores of each item and dividing this total score by the total number of items, the CVIs for the five experts ranged from 0.84 to 0.96, with a mean of 0.91; this met the CVI standard of 0.8 or above [37]. The items were also modified in line with experts’ opinions, which resulted in the final version of the questionnaire being completed.

Following this, we administered the PDI-MV to the recruited sample to complete the validity and reliability testing. One research assistant checked the list of cancer inpatients on a daily basis, screened patients for matched inclusion and exclusion criteria, and explained the research purpose and procedure to all recruited participants. The questionnaire was only administered after the participants had given their written informed consent. Participants’ autonomy was respected during questionnaire completion. Furthermore, participants were informed that they could stop filling in the questionnaire at any point. It took about 15 minutes to complete all questionnaires. Data were collected from February to May 2016.

## Data analyses

This study has adopted an exploratory approach, testing the PDI whether the Mandarin- speaking sample shows the same factor structure as previous samples with other language speakers. That makes exploratory factor analysis (EFA) an appropriate choice, more appropriate than confirmatory factor analysis (CFA). We performed data analysis using SPSS software version 19 (SPSS Inc., Chicago, IL, USA). Descriptive statistical analysis, including frequency, percentage, mean and standard deviation (SD), was performed for demographic and clinical variables. The concurrent validity of the PDI-MV was tested through correlations of this scale with the PHQ-9, DS-MV, and RSES. The internal consistency of the PDI was estimated by calculating Cronbach’s alpha for each factor. Item analysis included calculation of the means with SD, discriminatory power, skewness, and kurtosis.

The study used factor analysis to conduct the 25 items of PDI-MV with principal axis factoring extraction method and oblique rotation (promax) [38, 39]. The selection of the factors for rotation was based on the dual criteria of eigenvalues greater than 1 and an analysis of the screen plot [40]. The items with factor loadings were to examine both the highest and second highest factor loadings. In previous studies indicated the factor loadings 0.5/0.2 or 0.6/0.3 rule seems to constitute a norm. That is, an item is retained if its primary loading is greater than 0.5–0.6 and also if it’s second highest factor loading is smaller than 0.2–0.3 [41–43]. The Kaiser-Meyer-Olkin (KMO) was used to evaluate the sampling adequacy, and the Bartlett’s test of sphericity to test the study data derived from normal distribution with zero covariances [44]. Furthermore, a parallel analysis using Monte Carlo approach with 2000 randomly simulated datasets was performed, and the number of factor was determined using the eigenvalue calculated from our data and that from the random datasets [45]. For estimating the concurrent validity, Pearson’s correlation coefficient was used. All tests were reported on a two-tailed basis and p < 0.05.

## Ethical considerations

The study protocol received approval from the Institutional Review Board (IRB) of the recruitment institution (IRB number: 10411–003).

## Results

### Participant characteristics

In the study, 132 questionnaires were distributed while 125 were recovered; 7 due to did not meet study criteria, for an overall response rate of 94.7%. The participants were 66 males (52.8%) and 59 females (47.2%). The mean age of the participants was 57.76 years (SD = 11.61, range = 21–87), and the mean score on the PDI-MV was 38.91 (SD = 14.13, range = 25–93). The participants’ demographics and disease characteristics are shown in Table 1.

**Table 1:** Participants’ demographic and disease characteristics.

Characteristics		N = 125	%
Gender			
	Male	66	52.8
	Female	59	47.2
Marital status			
	Single	12	9.6
	Married	95	76.0
	Divorced	10	8.0
	Widowed	8	6.4
Education			
	≤ Elementary school	41	32.8
	Junior high school	26	20.8
	Senior high school	41	32.8
	> College	17	13.6
Employment			
	Yes	36	28.8
	No	89	71.2
Monthly household income (New Taiwan Dollar)			
	≤ 40,000	110	88.0
	> 40,000	15	12.0
Cohabitation status			
	Alone	10	8.0
	Live with family	115	92.0
Religious beliefs			
	Yes	108	86.4
	No	17	13.6
Tumor site			
	Digestive tract	54	43.2
	Breast	19	15.2
	Gynecologic	13	10.4
	Leukemia	11	8.8
	Head and neck	10	8.0
	Prostate	9	7.2
	Lung	7	5.6
	Others	2	1.6
Cancer stage			
	Stage I	20	16.0
	Stage II	23	18.4
	Stage III	37	29.6
	Stage IV	45	36.0
Clinical characteristics			
	Initial diagnosis	76	60.8
	Recurrence	49	39.2
Treatment			
	Curative	68	54.4
	Palliative	57	45.6

### Factor structure of PDI-MV

**Fig 1:**
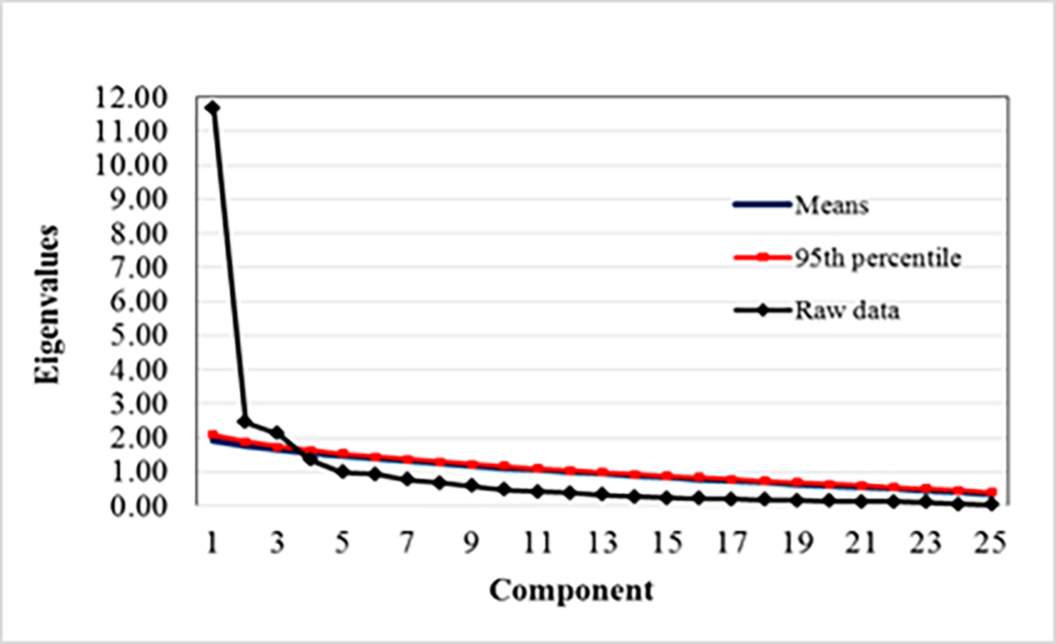
The number of component with scree plot and parallel analysis.

The study’s KMO measure of sampling adequacy was 0.88, Bartlett’s test of sphericity was *χ*^2^ (300) = 2833.60 (p < 0.001), indicating the correlations between items were significant for principal axis factoring. As a result of the factor analysis, four factors were explored on an examination of the factor scree plot. All four factors had eigenvalues of greater than 1 from the following factors at scree plot. In addition, the study adopted parallel analysis to analyze the number of component [45]. Table 2 presented the Monte Carlo information of parallel analysis. The result of scree plot with parallel analysis showed the number of factor was 4 (Fig 1). The four rotated factors are presented in Table 3. These factors were labeled as follows: existential distress, loss of support and sense of meaning, symptom distress, and loss of autonomy.

**Table 2:** The Monte Carlo information of parallel analysis.

Component	Means	95^th^ percentile	Raw data
1	1.93	2.08	11.68
2	1.77	1.88	2.45
3	1.65	1.74	2.12
4	1.55	1.64	1.36
5	1.46	1.53	0.99
6	1.38	1.45	0.94
7	1.31	1.37	0.76
8	1.24	1.30	0.67
9	1.17	1.23	0.58
10	1.11	1.16	0.48
11	1.05	1.10	0.42
12	1.00	1.04	0.39
13	0.94	0.99	0.32
14	0.89	0.93	0.27
15	0.84	0.88	0.23
16	0.78	0.83	0.22
17	0.74	0.78	0.19
18	0.69	0.74	0.19
19	0.64	0.69	0.16
20	0.59	0.64	0.14
21	0.55	0.59	0.13
22	0.50	0.55	0.12
23	0.46	0.50	0.10
24	0.41	0.45	0.05
25	0.35	0.40	0.04

N = 125, Variables = 25, percent = 95, Seed = 1953125.

**Table 3:** Factor analysis of PDI-MV.

No	Items		Item characteristics Factor Loadings						*h*^*2*^	Factor loadings			
		N	Mean	SD	*ri* (t-i)		Skewness	Kurtosis		Factor 1	Factor 2	Factor 3	Factor 4
**Existential distress (Cronbach’s alpha = 0.95)**													
19	Feeling that I don’t have control over my life	125	1.82	1.06	0.87		1.49	1.94	0.81	**0.99**	-0.15	-0.05	0.04
18	Feeling that I am a burden to others	125	1.79	0.94		0.73	1.19	1.11	0.75	**0.97**	-0.23	-0.18	0.17
16	Feeling I have unfinished business	125	1.78	0.99		0.68	1.67	2.87	0.61	**0.82**	-0.06	-0.05	-0.24
11	Feeling like I am no longer who I was	125	1.80	0.97		0.78	1.23	0.79	0.68	**0.78**	-0.02	0.03	0.07
14	Feeling that life no longer has meaning or purpose	125	1.53	0.84		0.75	1.66	2.51	0.71	**0.73**	0.24	-0.11	-0.06
23	Feeling like I am no longer able to mentally “fight” the challenges of my illness	125	1.54	0.75		0.71	1.24	0.78	0.70	**0.72**	-0.02	-0.06	0.29
17	Concern that my spiritual life is not meaningful	125	1.53	0.87		0.81	1.91	3.79	0.77	**0.71**	0.19	0.08	-0.10
20	Feeling that my illness and care needs have reduced my privacy	125	1.48	0.91		0.79	2.39	5.89	0.77	**0.68**	0.19	0.14	-0.21
24	Not being able to accept the way things are	125	1.44	0.68		0.73	1.41	1.30	0.64	**0.66**	0.06	-0.01	0.21
8	Worrying about my future	125	1.99	1.12		0.70	1.28	1.18	0.60	**0.64**	-0.08	0.24	-0.09
10	Not being able to continue with my usual routines	125	1.62	0.90		0.51	1.49	2.00	0.57	**0.60**	0.13	-0.003	0.38
7	Feeling uncertain about my illness and treatment	125	1.86	0.92		0.69	1.30	1.97	0.71	**0.59**	-0.19	0.42	-0.11
9	Not being able to think clearly	125	1.46	0.83		0.64	2.41	6.82	0.51	**0.59**	0.11	0.08	-0.15
13	Not being able to carry out important roles	125	1.53	0.80		0.67	1.64	2.83	0.64	**0.58**	0.04	0.04	0.30
12	Not feeling worthwhile or valued	125	1.51	0.93		0.71	2.11	4.21	0.64	**0.52**	0.33	-0.02	0.02
**Loss of support and sense of meaning (Cronbach's alpha = 0.84)**													
21	Not feeling supported by my community of friends and family	125	1.10	0.48	0.74		5.96	39.63	0.87	-0.15	**1.04**	-0.03	0.03
22	Not feeling supported by my health care providers	125	1.09	0.46	0.66		6.48	47.44	0.80	-0.09	**0.91**	-0.05	0.07
25	Not being treated with respect or understanding by others	125	1.24	0.59	0.77		2.81	8.30	0.71	0.30	**0.60**	-0.07	0.10
15	Feeling that I have not made a meaningful and lasting contribution during my lifetime	125	1.41	0.83	0.69		0.72	7.81	0.72	0.37	**0.51**	0.19	-0.16
**Symptom distress (Cronbach’s alpha = 0.83)**													
5	Feeling depressed	125	1.72	0.89	0.83		1.22	1.17	0.85	-0.04	-0.02	**0.96**	0.07
6	Feeling anxious	125	1.76	0.93	0.81		1.18	0.84	0.84	-0.01	-0.03	**0.93**	0.07
4	Feeling that how I look to others has changed significantly	125	1.49	0.75	0.50		1.75	3.74	0.52	0.17	0.17	**0.58**	0.03
3	Experiencing physically distressing symptoms	125	1.78	0.84	0.45		1.01	1.02	0.56	-0.10	-0.04	**0.51**	0.29
**Loss of autonomy (Cronbach’s alpha = 0.89)**													
1	Not being able to carry out tasks associated with daily living	125	1.38	0.82	0.70		2.59	6.93	0.86	-0.05	0.03	0.08	**0.92**
2	Not being able to attend to my bodily functions independently	125	1.26	0.69	0.77		3.12	10.59	0.83	-0.09	0.05	0.12	**0.88**
**Total Cronbach’s alpha = 0.95**			38.91	14.13			1.34	1.50					

### Item characteristics

The item characteristics of the PDI-MV are presented in Table 3. All 25 items showed adequate discriminatory power, with an *ri* (t-i) ranging from 0.45 to 0.87. Items 1, 2, 5, 6, 8, 11, 12, 14, 18, 20, 21, 23, 24, 25 showed *ri* (t-i) ≥ 0.70.

### Validity and reliability

Table 4 presents the inter-correlations of the PDI-MV factors. All four factors had significant correlations with each other (*r* = 0.16–0.71). Table 4 also shows the correlations among the PDI-MV subscales and other measures to determine the concurrent validity. All four factors were significantly correlated with the PHQ-9 (*r* = 0.25–0.54), DS-MV (*r* = 0.30–0.58), and the RSES (*r* = - 0.08 to—0.30).

**Table 4:** Inter-subscale correlations of the PDI-MV and concurrent validity measures.

Correlation matrix of subscales	PDI-MV		PDI-MV Subscales							
			Existential distress		Loss of support and sense of meaning		Symptom distress		Loss of autonomy	
	*r*	p	*r*	p	*r*	p	*r*	p	*r*	p
Existential Distress	0.98	< 0.01	1							
Loss of Support and Sense of Meaning	0.75	< 0.01	0.71	< 0.01	1					
Symptom Distress	0.76	< 0.01	0.62	< 0.01	0.40	< 0.01	1			
Loss of Autonomy	0.41	< 0.01	0.30	< 0.01	0.16	< 0.01	0.28	< 0.01	1	
**Concurrent validity measures**										
PHQ-9	0.54	< 0.01	0.49	< 0.01	0.25	< 0.01	0.50	< 0.01	0.38	< 0.01
DS-MV	0.58	< 0.01	0.57	< 0.01	0.30	< 0.01	0.47	< 0.01	0.32	< 0.01
RSES	-0.30	< 0.01	-0.27	< 0.01	-0.08	0.358	-0.29	< 0.01	-0.24	< 0.01

The Cronbach’s alpha was calculated for four factors using the items that loaded greater than 0.50 on each respective factor (Table 3). The Cronbach’s alpha for the overall PDI-MV was 0.95, and those for the existential distress, loss of support and sense of meaning, symptom distress, and loss of autonomy factors were 0.95, 0.84, 0.83, and 0.89 respectively (Table 3).

## Discussion

Dignity is a basic human right, and safeguarding the dignity of patients is a key responsibility of healthcare providers. In clinical practice, without a suitable tool for measuring dignity, patients’ dignity is hard to determine. This, in turn, makes it more difficult for healthcare providers to intervene in healthcare services when that dignity is threatened. Thus, we translated the PDI by Chochinov [15] into Mandarin and tested its validity and reliability. The study result found that the PDI-MV can help healthcare providers understand cancer patients’ dignity better and thereby provide effective intervention to preserve it.

The PDI was originally designed and validated in English [15], and translated into and validated in Persian [21], Italian [22], Spanish [23], and German [24], respectively. The Mandarin version was the sixth language version of the PDI. In terms of the participants except the Persian version were cardiac patients [21], our version and others were cancer patients [15, 22–24]. It indicated that the PDI could effectively measure the dignity level of cancer patients and would be applicable to cardiac patients.

“Regarding the factor structure of the PDI, the original English version has five factors [15], the Persian version [21], the German version [24] and the Mandarin of this study has four factors, the Spanish version has three factors [23] and the Italian version has one factor [22]. The items of the PDI that fell in the factors component across the six versions were somewhat different (Table 5). The possible explanations might be the differences of participant’s diagnosis, severity of disease, and duration of disease among the six versions. In this study, the participants were advanced and non-advanced cancer in-patients. In Spanish version, the participants were advanced cancer in-patients and out-patients [23]. In English version and German version, the participants were palliative cancer in-patients [15, 24]. In Italian version, the participants were non-advanced cancer out-patients [22]. In Persian version, the participants were cardiac in-patients [21]. However, we did not have data to verify our postulation, future studies using patients with similar characteristics across cultures are thus recommended to investigate the PDI structure again.

**Table 5:** Summary of the factorial structures of different versions of the PDI.

No	Items	Factor 1	Factor 2	Factor 3	Factor 4	Factor 5
1	Not being able to carry out tasks associated with daily living	I	S	E	G, M, P	
2	Not being able to attend to my bodily functions independently	I	S	E	G, M, P	
3	Experiencing physically distressing symptoms	E, I	S	G, M, P		
4	Feeling that how I look to others has changed significantly	I, S	E	G, M, P		
5	Feeling depressed	E, I, S	G, P	M		
6	Feeling anxious	E, I, S	G, P	M		
7	Feeling uncertain about my illness and treatment	E, I, M, S	G, P			
8	Worrying about my future	E, I, M, S	G, P			
9	Not being able to think clearly	E, G, M, I	P, S			
10	Not being able to continue with my usual routines	I, M	S	G, P		
11	Feeling like I am no longer who I was	I, M, S	E	G, P		
12	Not feeling worthwhile or valued	G, I, M, P	E	S		
13	Not being able to carry out important roles	G, I, M, P	E, S			
14	Feeling that life no longer has meaning or purpose	G, I, M, P, S	E			
15	Feeling that I have not made a meaningful and lasting contribution during my lifetime	G, I, P, S	M		E	
16	Feeling I have unfinished business	G, I, M, P, S			E	
17	Concern that my spiritual life is not meaningful	G, I, M, P	S		E	
18	Feeling that I am a burden to others	G, I, M	E, P, S			
19	Feeling that I don't have control over my life	G, I, M, P, S				
20	Feeling that my illness and care needs have reduced my privacy	I, M, P	G, S	E		
21	Not feeling supported by my community of friends and family	G, I, P	M	S		E
22	Not feeling supported by my health care providers	G, I, P	M	S		E
23	Feeling like I am no longer able to mentally 'fight' the challenges of my illness	I, M, S	G, P			
24	Not being able to accept the way things are	I, M, S	G, P			
25	Not being treated with respect or understanding by others	G, I, P	M	S		E

E = English version; G = German version; I = Italian version; M = Mandarin version; P = Persian version; S = Spanish version.

The Mandarin version of the PDI in this study, the Persian version [21] and the German version [24] showed four factors; the items of the factors component were similar (Table 5). In factor 1, the PDI of the Mandarin version contained 15 items (7, 8, 9, 10, 11, 12, 13, 14, 16, 17, 18, 19, 20, 23, 24); the Persian version contained 11 items (12, 13, 14, 15, 16, 17, 19, 20, 21, 22, 25); and the German version contained 12 items (9, 12, 13, 14, 15, 16, 17, 18, 19, 21, 22, 25). In factor 2, the Mandarin version contained 4 items (15, 21, 22, 25); the Persian version contained 8 items (5, 6, 7, 8, 9, 18, 23, 24); and the German version contained 7 items (5, 6, 7, 8, 20, 23, 24). In factor 3, the Mandarin version contained 4 items (3, 4, 5, 6); the Persian version contained 4 items (3, 4, 10, 11); and the German version contained 4 items (3, 4, 10, 11). In factor 4, the Mandarin version contained 3 items (1, 2); the Persian version contained 2 items (1, 2); and the German version contained 2 items (1, 2).

We used the PHQ-9, DS-MV, and RSES to test the concurrent validity of the PDI-MV. The results showed significant correlation between dignity and self-esteem, demoralization, and depression. Regarding the other versions of the PDI, significant correlations have been found with the PDI and measures of emotional distress, quality of life, physical symptom, spiritual wellbeing, anxiety, and depression [15, 21–24, 26]. Taken together, these findings indicate that the dignity level of patients is related to their psychological state. It is possible that patients suffer from psychological issues because of a low level of dignity, but if patients do not express this and healthcare providers do not notice, these psychological problems could progress and worsen their disease progression and even lead to a loss of the will to live. Thus, it is essential to regularly assess patients’ dignity using a valid and reliable tool such as the PDI.

In future studies, the PDI-MV could be expanded to various patient conditions for understanding patient's dignity, such as AIDS, hemodialysis, stroke, and long-term care, and an intervention protocol could be developed that could effectively improve patients’ dignity level.

## Limitations

The study have some limitations. First, in terms of the sample size in factor analysis, previous studies indicated the minimum necessary sample size ratio of *N* to the number of variables being analyzed, *p*. The *N*: *p* ration should be in the ratio of 3–6 [46, 47]. This study sample size *N*: *p* ratio was 5; it just achieved the minimum sample size requirement in factor analysis. Second, the participants in the study were not homogeneous and had various cancer conditions. Third, due to the participant’s practice, memory, physical and mental maturity could affect the test-retest reliability; in addition, cancer patient’s physical and psychological status were not suitable to be bothered repeatedly; consequently, we did not assess test-retest reliability. Furthermore, participants were recruited from only one medical center, so any generalizability of the study results should be cautioned.

## Conclusions

The results showed that the PDI-MV was significantly correlated with measures of self-esteem, demoralization, and depression in cancer patients. The PDI-MV can be useful in measuring cancer patients’ dignity and dignity-related distress, and it can be used in Mandarin-speaking countries. It is recommended that the use of this tool be evaluated for other non-cancer patients in Taiwan.

## Supporting information

S1 File Supporting information. Data of the study.
(XLSX)
